# Ultra-Small, High-Frequency, and Substrate-Immune Microtube Inductors Transformed from 2D to 3D

**DOI:** 10.1038/srep09661

**Published:** 2015-04-27

**Authors:** Xin Yu, Wen Huang, Moyang Li, Thomas M. Comberiate, Songbin Gong, Jose E. Schutt-Aine, Xiuling Li

**Affiliations:** 1 Department of Electrical and Computer Engineering; 2 Micro and Nanotechnology Laboratory, University of Illinois, Urbana, IL 61801; 3 International Institute for Carbon-Neutral Energy Research (I^2^CNER)

## Abstract

Monolithic on-chip inductors are key passive devices in radio frequency integrated circuits (RFICs). Currently, 70–80% of the on-wafer area of most RFIC chips is occupied by the sprawling planar spiral inductors, and its operation frequency is limited to a few GHz. With continuous scaling of the transistor technology, miniaturization and high frequency operation of inductors have become the bottleneck to meet future demands of wireless communication systems. Here we report on-chip self-rolled-up 3D microtube inductors with extremely small footprint, unprecedented high frequency performance and weak dependence on substrate conductivity. The serpentine metal strips are deposited on an oppositely strained silicon nitrides (SiN_x_) bilayer. After releasing from the sacrificial layer underneath, the metal/SiN_x_ layer is scrolled into a 3D hollow tubular structure by the strain induced unidirectional self-rolled-up technology. Compared to the planar spiral inductors with similar inductances and quality (*Q*) factors, the footprint of tube inductors is reduced by as much as two orders of magnitude, and the frequency at peak *Q* factor improves more than 5 times on doped substrates. The self-rolled-up 3D nanotechnology platform employed here, that “processes in 2D but functions in 3D”, is positioned to serve as a global solution for extreme RFIC miniaturization with improved performance.

With continuous innovations on transistors and interconnect architectures, complementary metal-oxide-semiconductor (CMOS) technology has been aggressively scaled beyond the soon to be in production 14 14nm node, allowing more and more transistors to be integrated on a chip. On the other hand, the progress of miniaturization of passive lumped devices has been severely behind, especially for inductors, the key component in radio frequency integrated circuits (RFICs)^1^,^2^. Metal conductors in planar spiral coil structures of the top interconnection layout, due to its compatibility with standard two-dimensional (2D) CMOS processing, are the commonly adopted. In order to achieve required inductance values, more and/or larger turns thus large footprint are often unavoidable due to weak coupling between the spiral wires. Furthermore, crosstalk between the radiated electromagnetic (EM) field and the substrate dramatically limits the inductor maximum working frequency and quality (*Q*) factor, especially when the substrate is heavily doped. Over the past several decades, significant progress has been made implementing advanced micro-electromechanical systems (MEMS) fabrication technologies to reduce the parasitic effects from the substrates^3^,^4^,^5^,^6^,^7^ and to scale down the dimension of planar spiral inductors^8^,^9^,^10^. These improvements include suspending or resistively isolating the spiral wires from the substrates or stacking vertically. However, the intrinsic problems of two-dimensional (2D) or quasi- three-dimensional (3D) open-ended structures inevitably lead to the tradeoff of the on-chip footprint and overall inductor electrical performances. In order to enhance the magnetic induction and reduce the substrate parasitic effects, the true 3D structures that can better confine the magnetic field and reduce large energy dissipation to free space and substrates are much more desirable. However, if conventional fabrication technologies (i.e. planar processing) are used to process 3D structures, issues such as mechanical stability, conformity, alignment, as well as cost, are difficult to address.

In this report, we experimentally demonstrate a new type of 3D tube inductor that is extremely small and with unprecedented high frequency performance and weak dependence on substrate conductivity. The 3D spiral tube inductor structure is enabled by the strain-induced Self-Rolled-Up Membrane (S-RUM) technology^11^,^12^,^13^,^14^,^15^,^16^,^17^,^18^,^19^, where strained thin film nanomembranes spontaneously roll up when released from their mechanical support. This is achieved by the removal of a sacrificial layer underneath the strained membrane, which triggers a net momentum to initiate and maintain the rolling as the embedded opposing biaxial strain is released. The resulted rolled-up structure is almost a perfectly round hollow cylinder, the diameter of which is determined by the material's mechanical properties and the thickness of each strained layer^20^,^21^,^22^. By employing a hierarchical SRUM platform with metal strips patterned in 2D and rolled up in multi-turns by a strained silicon nitride (SiN_x_) membrane^23^, combined with the novel unidirectional rolling process (see supplementary information for details), extremely compact 3D on-chip tube inductors are formed.

## Results

**Figure 1:**
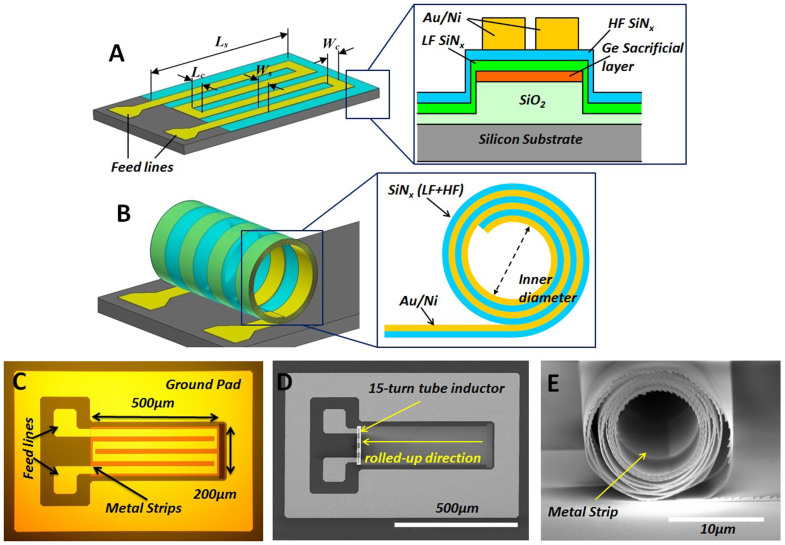
Schematic illustration of the 3D tube inductor design and images of fabricated devices before and after rolled up. (A) Schematic diagram of the 2D pattern of a tube inductor before rolled-up, with metal strips of width *W_s_* and length *L_s_* connected in series by connecting lines of width *W_c_* and length *L_c_*, and terminated with the feed-lines. The zoomed-in inset shows the cross-sectional structure and material stack. Note that current directions for all turns in the same strip are the same, leading to positive mutual inductance. For adjacent metal strips in the same or different planes, the current directions are opposite, however, the cancelling inductance is negligible when *L_c_* is long enough. (B) The corresponding rolled-up tube inductor. Inset shows the cross-sectional view of the hollow tube where the wall consists of multi-turns of SiN_x_ bilayer and metal (Au/Ni) strips. (C) Optical image of the before-rolled-up 2D inductor pattern with 4 metal strips (*W_s_* = 30 14μm and *L_c_* = 20 μm) and RF ground pads and feed-lines. The dimension of the metal strip pattern is 200 μm (width) × 500 μm (length) as indicated. (D) SEM image of the 4 metal strips tube inductor with 15 coiled turns, after the metal strip is self-rolled-up along the direction indicated together with the strained SiN_x_ membrane by the unidirectional rolling technique. (E) Cross-sectional SEM image of the 3D tube inductor with 15 coiled turns. The inner diameter (*ID*) is 10 μm and the outer diameter (*OD*) is 15 μm. The scale bar represents 10 μm.

Figure 1A and B illustrate the concept of multi-turns rolled-up inductors, using oppositely strained SiN_x_ bilayers as the rolling vehicle for conducting metal strips. Thin metal strips (Fig. 1A) with length *L_s_* and width *W_s_* connected by metal connecting lines with length *L_c_* and width *W_c_*, along with the two feedlines as the input/output ports of RF signals, are deposited and patterned on top of the SiN_x_ bilayer. Dual-frequency plasma enhanced chemical vapor deposition (PECVD) is used to deposit the SiN_x_ bilayer, i.e. the low frequency (LF), compressive layer and high frequency (HF), tensile stressed layer. By etching away the germanium (Ge) sacrificial layer, build-in stresses generate a net rolling moment to trigger the self-rolling process of the SiN_x_ bilayer and the metal strips, forming a multi-turns 3D tubular structure as shown in Fig. 1B. We emphasize that well-controlled unidirectional rolling is indispensable in order to produce tightly packed multi-turns in the tube inductors. This is realized by a novel sequence of patterning and deposition enabling coherent unidirectional rolling (Materials and Methods), which involves the use of a mesa and appropriate side-wall covering to ensure coherent tearing throughout the dynamic wet etching process (Fig. S1). Once the Ge sacrificial layer is completely removed, the rolling and tearing processing terminates abruptly and the rolled-up tube inductors are left sitting at the mesa edge held by the SiN_x_, as shown in Fig. 1B.

Geometrical control of tube inductors can be precisely realized with desired inner diameters and number of coiled turns, by pre-defining the residual stress, thin film thickness, metal pattern length along the rolling path. For all the structures studied in this work, the SiN_x_ bilayer thickness is 60 nm (30 nm HF + 30 nm LF) and the metal thickness is 100 nm Au/5 nm Ni. As an example, Fig. 1C shows the top view optical image of a four metal strips and fifteen coiled turns (4-strip/15-turn) tube inductor structure before it is rolled-up, where *W_s_* = 30 μm, *L_c_* = 20 μm, and *W_c_* = 10 μm. Fig. 1D and E are the corresponding SEM images showing the top and side views of the device after rolled-up. The inner and outer diameters are 10 and 15 μm, respectively. The on-chip footprint, which we define as the projection area on the substrate, is 200 × 15 μm^2^, i.e. 3000 μm^2^ or 0.03 mm^2^. Device layouts with other geometrical configurations are also shown in Fig. S2. Naturally, the on-chip footprint grows with the number of, width of, and spacing between the metal strips linearly, and increases only slightly with the number of coiled turns because of the ultra-thin SiN_x_ wall thickness. Compared to conventional 2D spiral inductors, the rolled-up inductor footprint is drastically reduced, by more than one order of magnitude for similar inductance values.

## Discussion

To examine the electrical performance of rolled-up inductors, two port scattering (*S*) parameters are measured in frequency range from 0.01 to 40 14GHz. Parasitic effects of the signal pads are removed by the “open-through” de-embedding procedure (Fig. S3). Measured *S_11_*-parameters from samples with different number of coiled turns and metal strips are plotted in the Smith Charts before and after de-embedding (Fig. S4). A lumped single-π equivalent circuit model illustrated in Fig. S5 is used to extract the effective inductance (*L*) and *Q* factor from admittance (*Y*) parameters, which are derived from the de-embedded *S* parameters.

**Figure 2:**
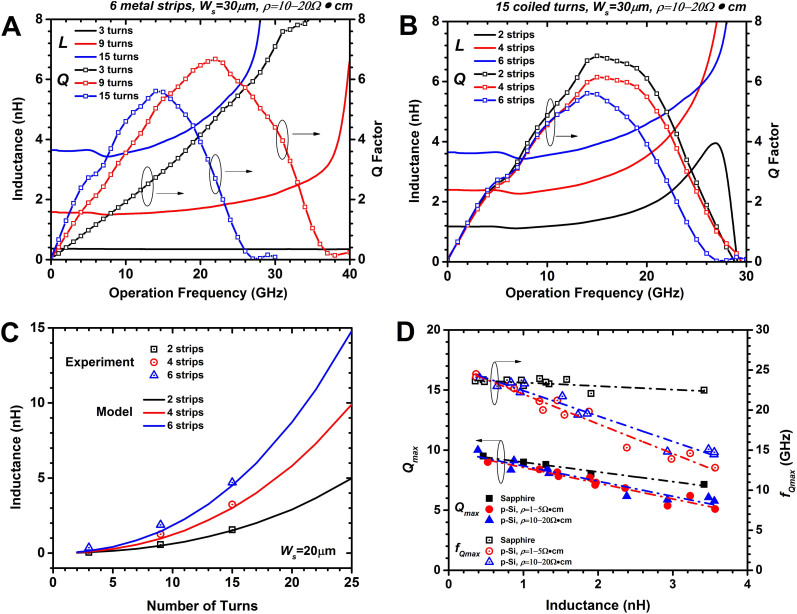
RF performance of tube inductors and their substrate immunity. (A) Measured inductances (solid lines) and *Q* factors (symbol lines) versus operation frequency for a series of tube inductors with 6 metal strips, *W_s_* = 30 μm, but different number (3, 9, and 15) of coiled turns on a *ρ* = 10 ~ 20 Ω·cm *p*-Si substrate. (B) Measured inductances (solid lines) and *Q* factors (symbol lines) versus operation frequency for a series of tube inductors with 15 coiled turns but different number of strips (2, 4, and 6). (C) Family of curves of inductance as a function of coiled turns for tube inductors with different number of strips (2, 4 and 6) with *W_s_* = 20 μm. The open symbols are experimental data, and solid curves are modeled data. (D) Experimental *Q_max_* and *f_Qmax_* of tube inductors with various configurations on three different substrates, plotted versus the corresponding inductance values. The dashed lines represent the linear fit of each data set obtained from respective substrates.

Fig. 2A and B show the extracted inductances and *Q* factors as the function of operation frequency for tube inductors with number of coiled turns and metal strips, respectively, on a *p*-Si substrate (*ρ* = 10 ~ 20 Ω·cm). As can be seen, the inductances of all devices hold steady over a wide frequency range before climbing up near *f_Qmax_* (the frequency corresponding to the peak *Q* factor). Just as the planar inductors, *Q* factors for rolled-up tube inductors increase almost linearly in the low frequency range, then reach the maximum value (*Q_max_*), after which they decrease with operation frequency until becomes zero at the self-resonant frequency (*f_0_*). As shown in Fig. 2A, for the 6-strip/15-turn and 6-strip/9-turn devices, the inductance remains at 3.6 and 1.6 nH until *f_Qmax_* at 12 and 22 GHz, respectively; for the 6-strip/3-turn device, the inductance remains constant (0.3nH) all the way to millimeter wave band (*f_Qmax_* is over 40 GHz, beyond the range of measurement). Compared to the planar spiral inductors with similar inductance values, the operation frequency for tube inductors are much higher than their planar counterparts^24^. Importantly, the inductance of the 6-strip tube inductor increases from 0.3, 1.6, to 3.6 nH, when the number of turns increases from 3, 9, to 15 turns, respectively, which is much faster than linear increase. Furthermore, in the low frequency range, the slopes of *Q* factors (the ratio of the effective inductances to direct current resistance) increase with the number of coiled turns. This suggests that, as more turns are formed, the rate of inductance increase is faster than that of resistance. The drop of *Q_max_* with increasing number of turns, on the other hand, can be attributed to the fact that more coiled turns correspond to larger inductance and more severe parasitic effects.

Larger inductances can also be achieved by connecting more metal strips in series. As shown in Fig. 2B, for the 15-turn devices, the inductance value is 1.2, 2.4, and 3.6 14nH for 2, 4, and 6 metal strips spaced by 20 μm connecting lines, respectively, showing a precise proportional inductance increase with the number of strips. Note that this perfect proportionality observed here only holds truth when metal strips are separated sufficiently far apart that the canceling mutual inductance between strips (with opposite current flow directions) is negligible. In contrast to Fig. 2A, the *Q* factors, as more metal strips are connected serially with more contact area on the substrate, show nearly identical slopes at low frequency, indicating that the rate of increase of inductance and resistance are almost identical. Also, comparing to the Fig. 2A, *Q_max_* and *f_Qmax_* do not change much with the number of metal strips, it can be inferred that the parasitic effects from the substrate are weaker than those between coiled turns.

In the Fig. 2C and Fig. S6, the experimental (open symbols) and calculated (solid curves) inductances as functions of the number of coiled turns and metal strips width are plotted with number of metal strips. The calculated data are derived from our physical model proposed previously^25^ and details of comparison between experimental and model results are demonstrated in Fig. S7. In the Fig. 2C and S6, excellent agreements between the calculated and experimental inductance values are found in all devices, and the slight discrepancy should be attributed to the additional inductances of connecting lines which are not considered in the model. Physically, the effective inductance of a tube inductor can be comprehended as two parts, the constructive mutual inductance between all coiled turns and the cancelling mutual inductance between adjacent metal strips. Obviously, for a planar straight wire, only self-inductance occurs when RF current flows through it; but once the wire is coiled into a 3D spiral structure with very little gap between each layer, the magnetic flux is expected to be confined into the inner side of hollow low attenuation air core structures. So the constructive mutual inductance between each metal layer becomes more predominant, owing to the strong EM field coupling. Therefore the tube inductors have much higher inductance than planar spiral structures with same footprint. As shown in Fig. 2C, the inductance increases exponentially as a function of number of coiled turns, indicating that the mutual inductance is strongly enhanced between turns in the 3D tube inductor structure. This is a result of the close electromagnetic field coupling between turns that have identical current flow directions and are separated only by the 60 nm SiN_x_ bilayer membrane. On the other hand, different from the macroscopically solenoid inductor, in the tube inductor, the current flowing directions in the two adjacent strips are opposite, which introduces a cancelling mutual inductance between them. Based on our calculation results, when two adjacent strips are spaced (*L_c_* in Fig. S8) far enough, the cancelling inductance can be neglected. In Fig. 2C, the length of connecting line is 20 μm, which has made the cancelling inductance smaller than 1% of inductance for one spiral metal strip with 15 turns. Therefore, when more metal strips are connected in series, the inductance can increase proportionally to the number of strips, providing another way to increase inductance without changing the number of turns.

For an inductor, only the energy stored in the magnetic field is desired, and any energy stored in the electric field induces parasitic resistance and capacitance. In the series branch of the equivalent circuit model of a tube inductor, both ohmic loss (DC range) and skin effect (RF range) should be minimized. Based on DC measurement results, 100 14nm thick Au film under our optimized deposition condition has a resistivity that is 1.3 times larger than its bulk value. So, at 40 GHz high frequency, the skin depth is 420 nm. Therefore, the skin effect can be ignored and the tube inductor resistance is almost frequency independent up to millimeter wave band. In the shunt branch, the parasitic capacitances include the crosstalk capacitance (*C_c_*) between adjacent turns and the substrate parasitic capacitance (*C_s_*), which consists of SiO_2_ capacitance (*C_ox_*) and doped Si capacitance (*C_Si_*). In particular, *C_c_* cannot be neglected between turns because of the gap between each turn is only 60 nm thick of SiN_x_ membrane. As shown in Fig. S9, comparing the open-ended device structure of planar spiral inductors, considerable electromagnetic (EM) field penetrates and dissipates into the substrate. In contrast, the 3D hollow tubular structure of the tube inductor confines most of EM field inside the tube, with little dissipation into the substrate. Therefore the *C_s_* plays a secondary role relative to *C_c_*. Indeed, based on the extracted parameters and calculated results for a *ρ* = 10 ~ 20 Ω·cm *p*-Si substrate, the overall inter-turns *C_c_* is tens of femto-faradays (*f*F), and the combined *C_ox_* and *C_Si_* is only a few *f*F (Table S1).

In order to comprehensively evaluate the substrate effect, a large set of tube inductor devices with identical material but different geometrical configurations are fabricated on three different kinds of substrates, *ρ* = 1 ~ 5 Ω·cm *p*-Si and *c*-plane sapphire, in addition to the *ρ* = 10 ~ 20 Ω·cm *p*-Si substrate used previously. Plotted in Fig. 2D are the *Q_max_* and *f_Qmax_* as the function of inductances for the three types substrates. The frequency dependence of inductances and *Q* factors for the 4-strip/15-turn device can be found in Fig. S10, where the inductance values are exactly the same on all substrates. In the Fig. 2D, for all substrates, *Q_max_* and *f_Qmax_* decrease with the inductance, and the slopes of the decrease (the slopes of the linear fittings) vary with substrate conductivities, especially, sapphire showing the slowest degradation, as expected. For the two doped silicon substrates studied, the linear fits of *Q_max_* and *f_Qmax_* show almost the same slopes and near identical values for the entire inductance range, from 0.4 to 3.5 nH. Their deviations from the sapphire curve become larger with increasing inductance; however, the substrate effect is much less severe for the tube inductors compared to planar counterparts. As an example, in Fig. 2D at the 3.5 nH, the maximum degradation of *Q_max_* is only 30% and *f_Qmax_* degradation is 50%; in contrast to 150% degradation of *Q_max_* at 3.5 nH and 100% degradation of *f_Qmax_* at 5 nH for planar spiral inductors^26^. It is notable that, to achieve the same inductance values and with comparable *Q* factors, the *f_Qmax_* of tube inductors are much higher than that of planar inductors on the same substrates, including doped substrates with low resistivity. For example, at 2.1 nH, on a sapphire substrate, *f_Qmax_* achieved with the tube inductor is 23 GHz compared to 5.6 GHz for a typical planar inductor^26^ and on the *ρ* = 10 Ω·cm *p*-Si substrate, the *f_Qmax_* enhancement is from 3.5 GHz for a representative planar inductor^26^ to 19 GHz for the tube inductor. In summary, compared to its planar counterpart, the tube inductors demonstrated excellent substrate immunity and high frequency performance, which can be attributed to its extremely small on-chip footprint and much better confinement of EM field to prevent dissipation to lossy substrates.

**Figure 3:**
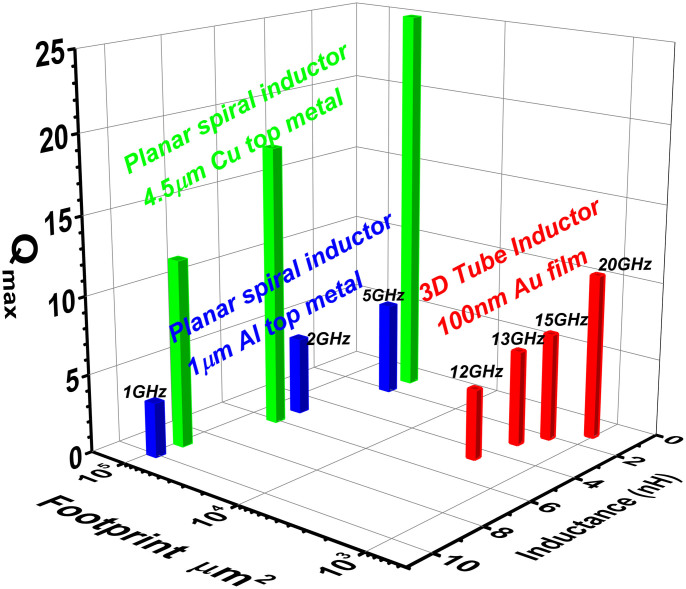
Benchmark 3D plot. Inductance, footprint, and *Q_max_* of rolled-up 3D tube inductors (represented by red pillars) are plotted together with those for planar spiral inductors used in the 0.18 μm and 32 nm node CMOS technology. The marked frequencies correspond to *f_Qmax_* of the inductors. The metal type and thickness for the tube inductor and the two generations of planar inductors are as indicated. The substrate resistivity is *ρ* = 1 ~ 5 Ω·cm *p*-type for the red and blue pillars and *ρ* = 10 Ω·cm *p*-type for the green pillars.

For a comprehensive overview of all the metrics discussed, Fig. 3 benchmarks the footprint and RF performance (*L*, *Q_max_*, *f_Qmax_*) of the 3D tube inductors, with the state-of-the-art planar spiral inductors used in the 0.18 14μm^24^ and 32 nm node^27^ CMOS technology, where much thicker metal layers (1 μm Al and 4.5 μm Cu, respectively) were used. For the same inductance values achieved, the corresponding *f_Qmax_* is much higher than that of planar inductors on substrates with similar resistivity. Most remarkably, the rolled-up inductors only occupy 1–10% or less of the on-chip area in comparison. Clearly, once the strained thin film is rolled-up, there are voids left behind on the sample, which does not reduce the overall footprint at the top level layout. One approach to realize high density assembly and integration of rolled-up passive devices is to use the transfer printing process, which can extract the rolled-up tube from the native substrates and transfer to a receiving substrate in desired pattern and density. Preliminary success of transferring the S-RUM tube array off their native substrates has already been demonstrated previously^14^ and further development is underway. On the other hand, by rolling up the inductors, most of the substrate area could potentially be saved for CMOS devices, resulting in a compact circuit layout. This is in contrast to the circuit layout with planar spiral inductors, where the close proximity of inductors and active devices is not possible because of the strong unwanted EM field coupling between them.

In conclusion, we have successfully demonstrated on-chip self-rolled-up 3D tube inductors. This is achieved by unidirectional S-RUM nanofabrication technique, employing the build-in stress in the SiN_x_ bilayer hierarchically integrated with 2D patterned ultra-thin metal strips. The rolled-up tube inductors have shown record inductance/area density, due to the strong mutual coupling effect, which enables an extremely small footprint while maintaining the same inductance. The strong confinement of electromagnetic field within the tube prevents it from dissipating into the substrate. As a result, even on heavily doped silicon substrates, all devices show high self-resonant frequencies, beyond the range achievable by any reported planar or quasi-3D counterparts of the same inductance, highlighting the unprecedented immunity to substrates. By replacing Au with lower resistivity metal such as Cu, Ag, and possibly graphene, *Q* factors can be increased to be suitable for high performance RFICs. This work establishes S-RUM as a new paradigm that processes like 2D and functions like 3D for other 3D miniaturized functional devices. The ability to control the size, pitch, and orientation in plane, as well as vertical stacking, will enable more complex circuit designs simply using conventional optical lithography. In particular, S-RUM passive electronics can lead to a new kind of RFICs that is ultra-small, light, and highly integratable for applications in portable and wearable electronics in high frequency band.

## Methods

To fabricate the 3D rolled-up tube inductor (figure. S1), a 1 μm thick silicon dioxide (SiO_2_) was grown by wet thermal oxidation to electrically insolate from the *p*-type silicon (*p*-Si) substrate (Note 1 μm thick PECVD SiO_2_ was deposited on the sapphire). On the SiO_2_, a 20 nm Germanium (Ge) sacrificial layer was deposited by electron beam evaporation, and it helps to fix and retain the residual stress of all strain layers. The rectangular mesas were defined by optical lithography and reactive ion etching (RIE). Subsequently, the 60 nm strained SiN_x_ bilayer was deposited by dual-frequency plasma enhanced chemical vapor deposition (PECVD) to cover the entire sample, which is composed by a 30 nm 380 KHz low frequency (LF) SiN_x_ and a 30 nm 13.56 MHz high frequency (HF) SiN_x_. Technically, during the deposition, LF RF power induces a compressive strain into the SiN_x_ thin film, which can be attributed to the excess Si atoms embedded into Si-N and Si-H bonds of SiN_x_ by bombardment effect. On the other hand, the HF RF power introduces a tensile strain into the SiN_x_ membrane because of fewer Si atoms and lower density compared to standard stoichiometric ratio Si_3_N_4_ (*S1*). In our case, the residual stress of LF and HF SiN_x_ thin film are −900 MPa and +300 MPa, respectively. Then, the 100 nm gold (Au)/5 nm nickel (Ni) serpentine metal lines are deposited on SiN_x_ bilayer by electron beam evaporation and metal lift-off technology, with +300 MPa and +600 MPa residual stresses, respectively. Subsequently, a deep trench at one side of mesa is defined by optical lithography and RIE, which is down through to SiO_2_ layer. At last, the sample undergoes a wet etching in hydrogen peroxide at 90°C.

## Supplementary Material

Supplementary Information Supplementary Information
