# Supplementary material for: Ultra-Small, High-Frequency, and Substrate-Immune Microtube Inductors Transformed from 2D to 3D
Source: Sci Rep. 2015 Apr 27;5:9661. doi: 10.1038/srep09661 (PMC5386192; doi:10.1038/srep09661)
Supplement: Supplementary Information [file srep09661-s1.pdf]

Supplementary Information

Ultra-Small, High-Frequency, and Substrate-Immune Microtube Inductors Transformed from 2D to 3D

Xin Yu, Wen Huang, Moyang Li, Thomas M. Comberiate, Songbin Gong, Jose E. Schutt-Aine, Xiuling Li*
Department of Electrical and Computer Engineering, Micro and Nanotechnology Laboratory,
University of Illinois, Urbana, IL 61801
*xiuling@illinois.edu

Supplementary Materials:
Figures S1–S10
Table S1, S2
References (*S1 –S5*)

1. The Detailed Step-by-step Processing Flow

1) Silicon wafer preparation, standard RCA clean,
   $NH_4OH:H_2O_2:H_2O=1:1:5$, at 80℃, 10min,
   $HF:H_2O=1:100$, at room temp, 1min,
   $HCl:H_2O_2:H_2O=1:1:5$, at 80℃, 10min,
   DI water raise, N_2 drying.
2) Wet Thermal Oxidation 1μm SiO_2, at 1150℃, 2hours.
3) 20nm Ge is deposited by electron beam evaporation, with 0.5Å/sec rate.
4) Define the rectangle tube patterns,
   Spin on positive photoresist (AZ5214E),
   Soft bake 1min at 110℃,
   Exposed by 365 nm UV lithography with optical photo mask,
   Develop in MIF 917 developer,
   O_2 descumming 2min for PR residual removal.
5) Reactive Ion Etching by CF_4,
   Etching through Ge and 50nm down to SiO_2,
   Acetone, Methanol, Isopropanol strip PR,
   O_2 descumming 2min for PR residual removal.
6) Dual-frequency STS-PECVD (Surface Technology Systems plc.) deposition, at 300℃ platen, 240℃ showerhead,
   30nm LF SiN_x deposition, 380KHz RF power, 20W, $SiH_4:NH_3=1:1$, 300mT,
   30nm HF SiN_x deposition, 13.56MHz RF power, 20W, $SiH_4:NH_3=4:5.5$, 900mT.
7) Au/Ni strips deposition,
   Spin on image reversal photoresist (AZ5214E-IR),
   Soft bake 1min at 110℃,
   Exposed by 365 nm UV lithography with optical photo mask,
   Reversal bake 1min at 110℃,
   Flood exposure,
   Develop in MIF 917 developer,
   O_2 descumming 2min for PR residual removal,
   5nm Ni, followed by 100nm Au deposition by electron beam evaporation, with 0.5Å/sec and 1.5Å/sec rate, respectively,
   Metal lift-off in AZ 400T stripper at 80℃ 10min.
8) Define the deep trench,
   Lithography is as same as step 7 with different photo mask.
9) Reactive Ion Etching by CF_4,
   RIE processing is as same as step 5.
10) Lateral etching for tube inductor unidirectional scrolling,
   Etchant, H_2O_2:citric acid =25:1, at 90℃ 2hours for 15 coiled turns tube inductors,
   Citric acid is citric acid monohydrate: H_2O=100g:200mL, stirred 12hours.

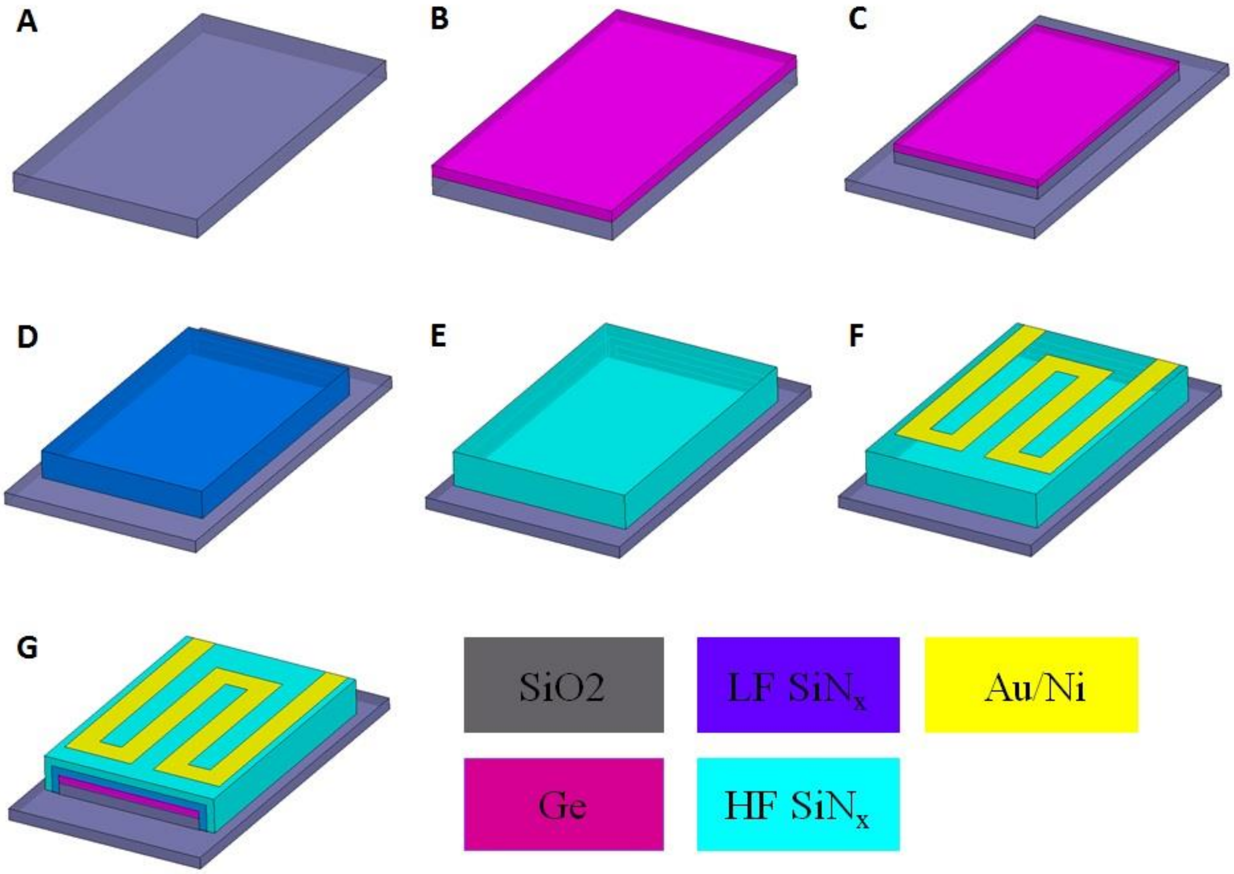

Figure S1: Schematic diagrams of the 3D rolled-up tube inductors fabrication processing.
(A) 1µm silicon oxide layer is grown by wet thermal oxidation (Si substrate is not shown).
(B) Deposit 20nm Ge sacrificial layer.
(C) Mesas are defined by optical lithography and reactive ion etching (RIE).
(D) Deposit 30nm LF SiN_x thin film to cover the whole mesas.
(E) Deposit 30nm HF SiN_x thin film on the top of LF SiN_x.
(F) 5nm Ni and 100nm Au stripes are deposited on the top of the HF SiN_x of mesas.
(G) A deep trench along one side of mesa is defined by optical lithography and RIE.

2. Unidirectional Rolled up Mechanism and Technology

Unidirectional rolling up is indispensable for multi-turns tube inductors. When the removal of the exposed sacrificial layer initiates, the opposing built in strain of the bilayer generates a net momentum - the LF SiN_x prefers to expand and HF SiN_x and Ni/Au to shrink- to lift all the layers up from the substrate. As the Ge is etched away continuously, the layers wrap down to substrate surface and scroll into a spiral cylinder tube architecture. Although the biaxial stresses of amorphous SiN_x are isotropic, Ge is only removed away from one side by etchant, and other three sides are covered and protected by SiN_x bilayer. Therefore, the scrolling process just happens from the deep trench direction, as shown in fig.1 C and D. On another aspect, during the sacrificial layer is etched away, the net momentum also tears apart the SiN_x bilayer at the sidewalls of Ge mesas. The saw-tooth patterns on the edges of mesa and tube are the direct evidence, as shown in Fig.1E. This dynamic tearing process ensures unidirectional multi-turns rolling throughout the wet etching. Once the Ge is removed totally, the rolling and tearing processing terminates, immediately, and the tube inductors are hold by SiN_x bilayer and stand on the substrate surface.

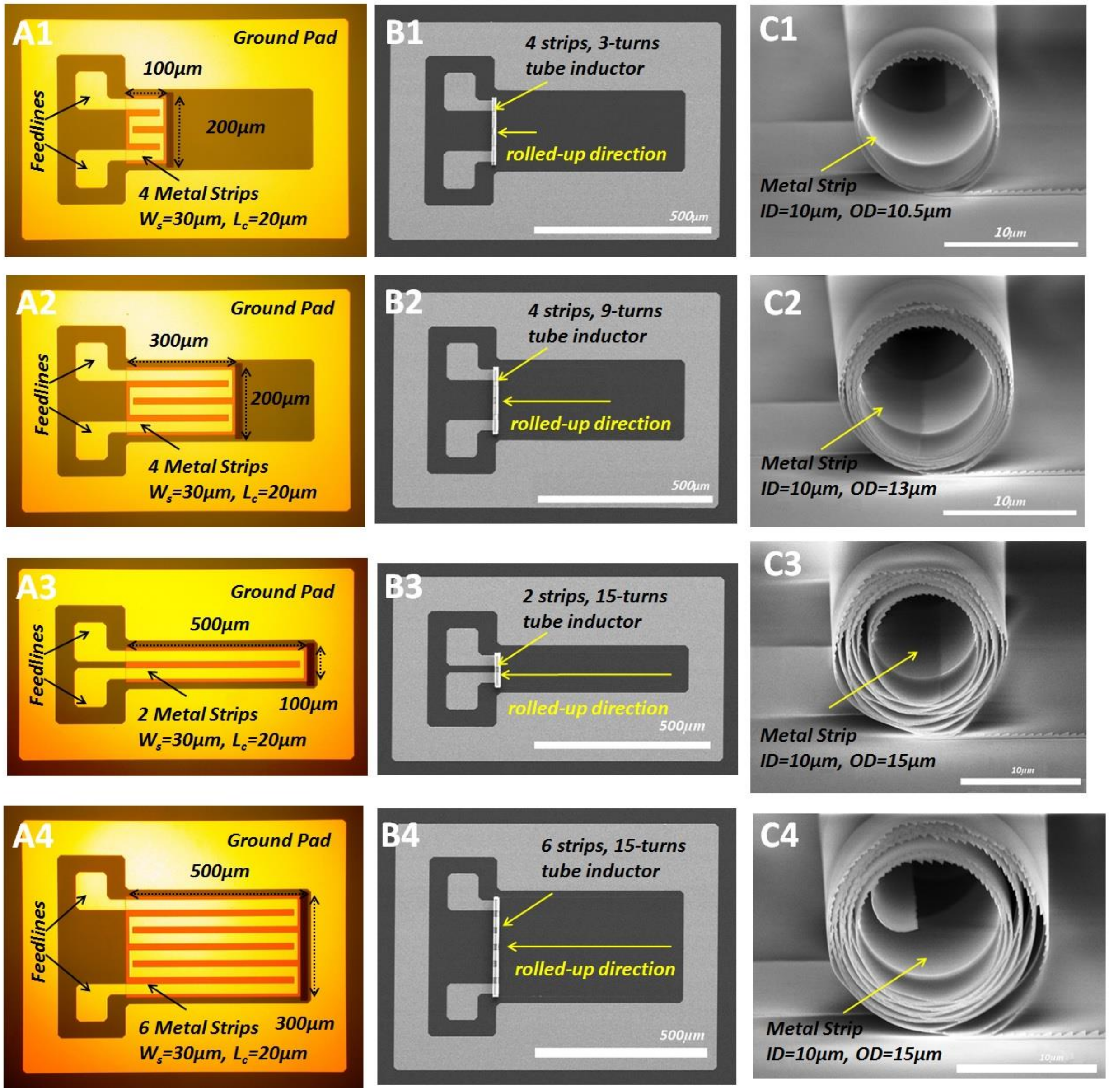

Figure S2: The optical microscopy and SEM images of self-rolled-up tube inductors with different structural configurations. A1-C1 show 4-strip/3-turn inductor; A2-C2 show 4-strip/9-turn inductor, A3-C3 show 2-strip/15-turn inductor, A4-C4 show 6-strip/15-turn inductor. (A1-A4) the top view of 2D patterns before rolled up by optical microscopy. Longer patterns induce more coiled turns and more metal strips need wider patterns. (B1-B4) the top view of 3D tube inductors by SEM after the Ge layer is released thoroughly. (C1-C4) the cross sectional view of rolled up tube inductors. The outer diameter increases with number of coiled turns, along with the constant inner diameter.

3. De-embed Methodology

The open-through de-embedding procedure is adopted to calibrate out the parasitic capacitance and resistance from the feedlines (*S2*, *S3*). The contact fixture is designed as shown in figure S3 (A-C), and the lumped equivalent circuit model is constructed to represent the physics of parasitic effects, as shown in the insets. As the RF measurement goes up to 40 GHz, feedlines are designed as short as possible to minimize the distribution effect. As shown in inset of figure S3 (A), the admittance π-network is used to model the capacitive effects between the contact pads and the surrounding environment including the substrate and RF ground. Series connected impedance network is used to model the resistance and inductance of the feedlines.

Mathematical procedure to do the open-through de-embedding is shown in Figure S3 (D-F). In the first step, the admittance π-network (open pattern) is abstracted from the original data (DUT). Then, the parasitic resistances and inductances (Z_1 and Z_2) can be calculated by step 2 (Figure S3 E). Finally, the real performance of DUT can be obtained by step 3 (Figure S3 F).

In the Figure S4 (A-D), the embedded and de-embedded S_{11} parameters of tube inductor *p*-Si substrate (ρ=10-20 Ω▪cm) are plotted in smith charts. (A and C) are for 3, 9 and 15 turns tube inductors with 6 metal strips. (B and D) are for 2, 4, 6 strips with 15 coiled turns tube inductors.

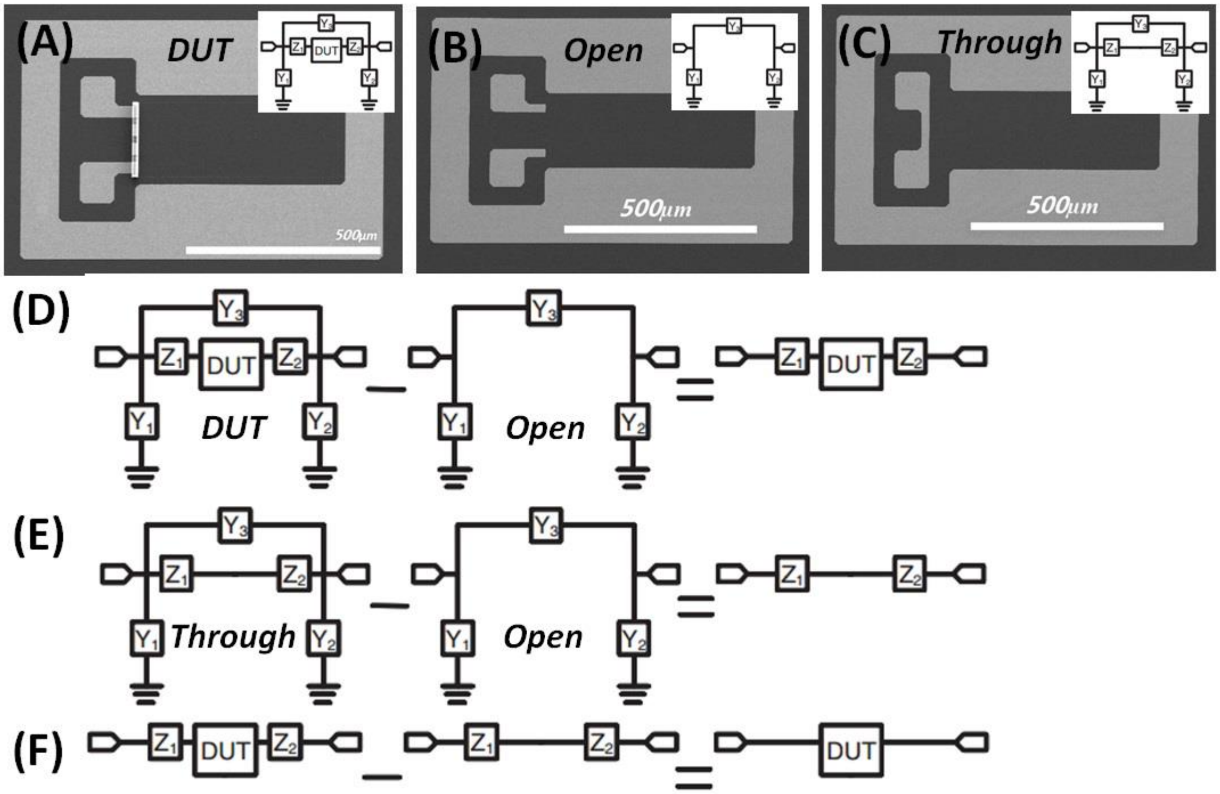

Figure S3: The open-through de-embedding methodology. (A-C), the schematic view of patterns with the device under test (DUT), without the DUT (open pattern) and two feedlines are connected directly (through pattern). Corresponding lumped equivalent circuits are constructed to model the RF performance of each pattern (insets). The narrow branches of feedlines are bended 90 degrees to avoid using additional lines to connect the two feedlines together. (D-F), Mathematic procedure for the open-through de-embedding, (D) Subtract measured data of open pattern from raw data with DUT, (E) Subtract measured data of open pattern from data of through pattern, (F) Subtract data obtained from step (E) from data of from step (D).

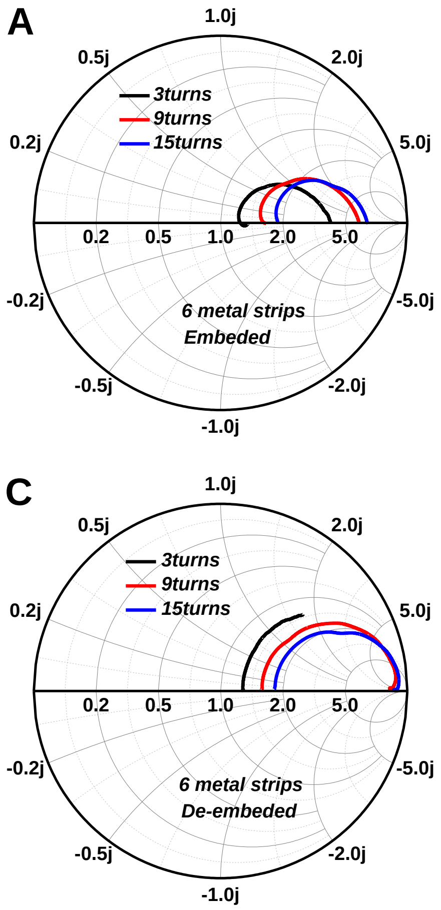

Figure S4: The smith charts of measured S_{11} parameters of tube inductors with different structures on a *p*-Si substrate (ρ=10-20Ω▪cm). (A and B) are before de-embedding, and (C and D) are after de-embedding. (A and C) Black, red and blue curves for the 3, 9 and 15 coiled turn tube inductors with 6 metal strips and W_s=30μm. (B and D) Black, red and blue curves for the 2, 4, 6 metal strip tube inductors with 15 coiled turns and W_s=30μm.

4. Equivalent Circuit and Parameter Extraction

After de-embedding, the inductor parameters were extracted based on the π equivalent circuit, as shown in Figure S5. Detailed descriptions of the physical equivalent model can be found in Reference *S4*. Total inductance and resistance of the tube inductor are represented by serial inductance (L) and serial resistance (R), respectively. The sum of all overlap direct coupling between different turns of metal strip lines represents the inter-turn distribution capacitance (C_c). The substrate parasitic capacitance (C_s) represents the capacitive coupling from coil spiral strip line and connecting line of inductor to ground. Therefore, from the Y parameters admittance matrix, the measured two-port inductor parameters are determined as follows. All the extracted parameters are listed in Table S1.

$$L = \frac{Im\left(-\frac{1}{Y_{12}}\right)}{\omega} = \frac{Im\left(\frac{1}{Y_s}\right)}{\omega} \quad (1)$$

$$Q = \left|\frac{Im(Y_{11})}{Re(Y_{11})}\right| = \left|\frac{Im(Y_p + Y_s)}{Re(Y_p + Y_s)}\right| \quad (2)$$

$$R = Re\left(-\frac{1}{Y_{12}}\right) = Re\left(\frac{1}{Y_s}\right) \quad (3)$$

$$C_s = \frac{Im(Y_{11} + Y_{12})}{\omega} = \frac{Im(Y_p)}{\omega} \quad (4)$$

$$C_{crosstalk} = \frac{\sqrt{[(N_cL_0^2\omega^2+N_cR_p^2-2LR_p\omega)\times(N_cL_0^2\omega^2+N_cR_p^2+2LR_p\omega)]}-N_cR_p^2-(N_cL_0^2\omega^2-2LL_0\omega^2)}{2(LL_0^2\omega^4+LR_p^2\omega^2)} \quad (5)$$

N_c=number of metal strips
R_p=R(value at 0.01GHz)/N_c
L_0=L(value at 0.01GHz)/N_c
$\omega=2\pi f$

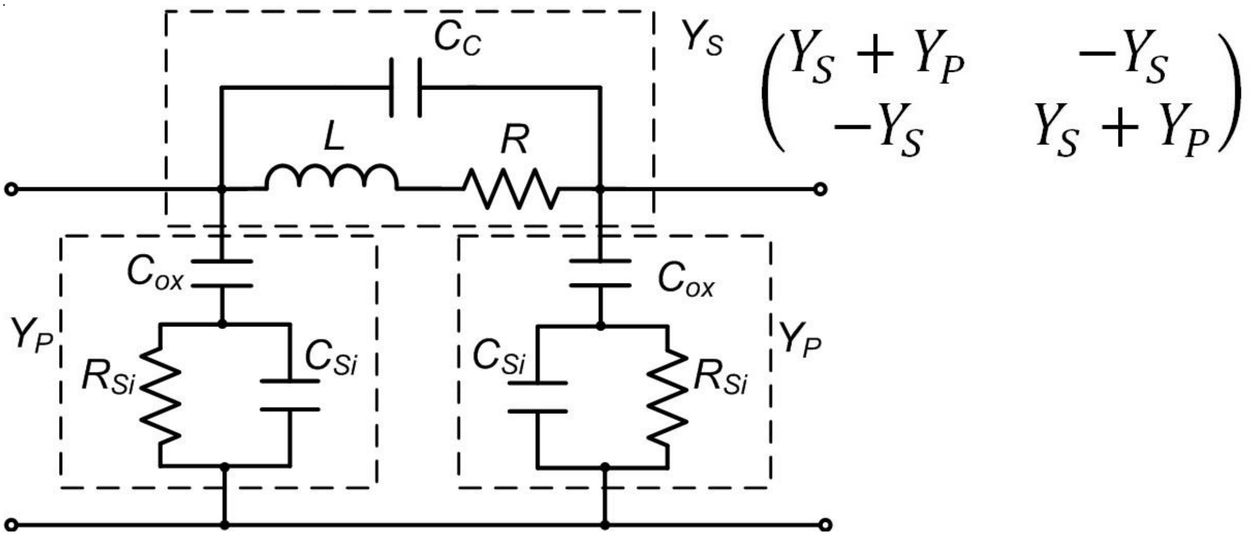

Figure S5: the π lumped equivalent circuit for the lumped parameters extraction, which is based on the physical structure of tube inductor on doped Si substrate.

Table S1: Extracted parameters based on the equivalent circuit model for various device structures.

Device structure (W_s=30µm.)	L (1GHz)	R (1GHz)	C_c (20GHz)	C_s (20GHz)
3turns, 6strips	0.3 nH	11.3 Ω	55 *f*F	4.5 *f*F
9turns, 6strips	1.6 nH	23.8 Ω	39 *f*F	5 *f*F
15turns, 6strips	3.6 nH	34.3 Ω	18 *f*F	5.5 *f*F
15turns, 4strips	2.4 nH	24 Ω	26 *f*F	2.2 *f*F
15turns, 2strips	1.2 nH	11.1 Ω	47 *f*F	1.1 *f*F

5. Effect of metal strip width on inductance

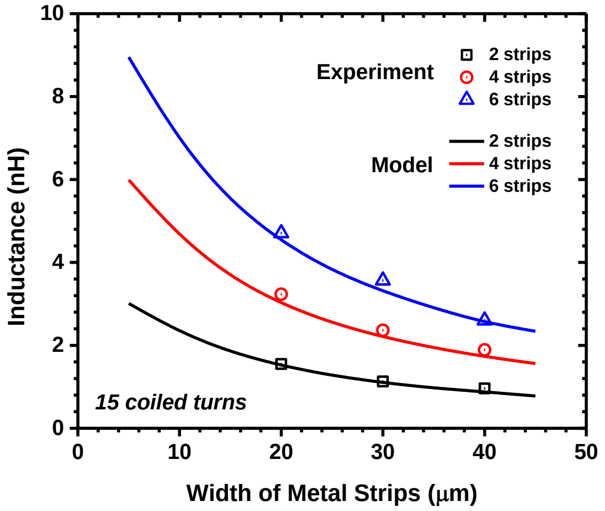

Figure S6: The family curves of *L* as a function of the width of metal strips, for devices with different number of metal strips. The open symbols are experimental data, and solid curves are derived from the physical model (*S4*).

As expected, the inductance decreases with the width of metal strips, because the wider metal strip generates the lower current density and weaker average magnetic flux density.

6. Physical Modeling and Comparison with Experimental Results

Employing the our developed physical model (*S4*), the measured effective inductances versus frequency from 0.01GHz to 40 GHz are plotted and compared with modeled curves. The device structural parameters, such as inner/outer diameter, metal strips width, number of strips and turns, are input into the model, which are as same as that of the real inductors. Tube inductances with the functions of coiled turns and metal strips are compared in the figure S6 A and B, respectively. Good agreement between the measured and modeled data can be found, which validates the high accuracy and reliability of the physical model on predicting inductance of the tube inductors. Therefore the model can calculate inductance values with more turns.

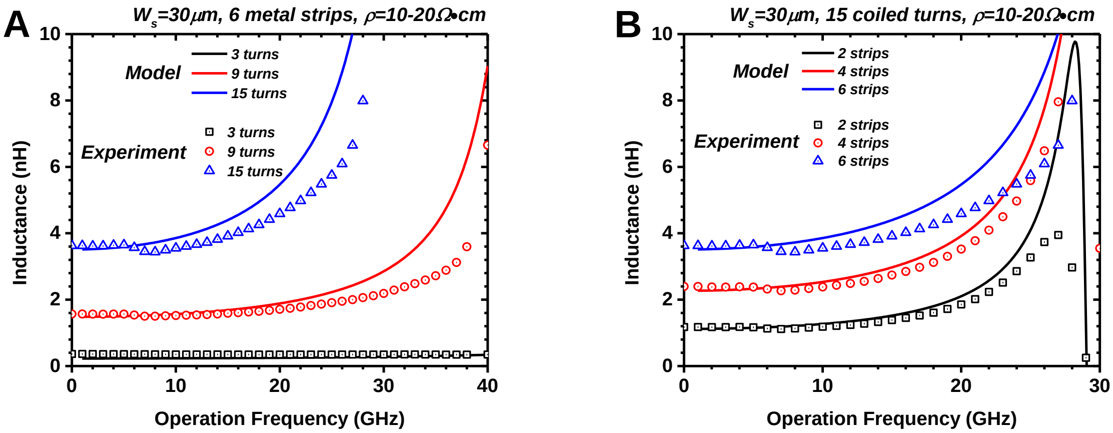

Figure S7: Inductance comparison between the measured and modeled data (*S4*). (**A**) Experimental (open symbols) and modeled (solid curves) inductances for the 3, 9 and 15turns tube inductors with 6 metal strips. (**B**) Experimental (open symbols) and modeled (solid curves) inductances for the 2, 4 and 6 strips tube inductors with 15 turns.

7. Effect of separation between the adjacent metal strips on cancelling mutual inductance.

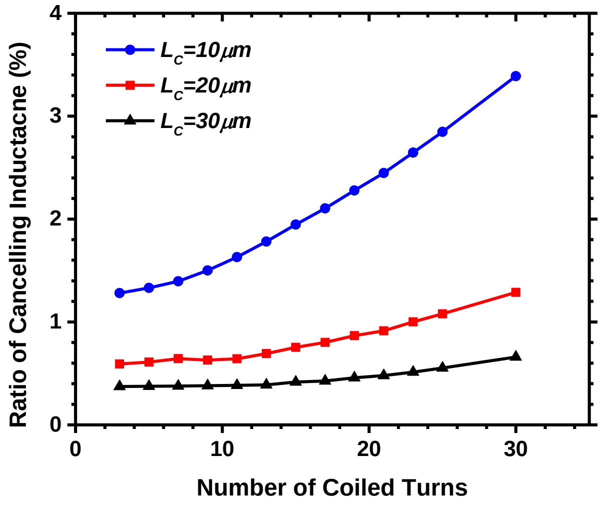

Figure S8: The family curves of ratio of cancelling inductance as the function of number of coiled turns with different L_c

Based on the physical model of tube inductor (*S4*), the inductance of one strip and two strips connected by L_c can be calculated. If the ratio of cancelling inductance is defined as,

$$Ratio\ of\ cancelling\ inductance = \frac{2 \times one\ strip\ inductance - inductance\ of\ two\ strips\ connected\ by\ L_C}{inductance\ of\ two\ strips\ connected\ by\ L_C} \times 100\%$$

it could be plotted to the number of coiled turns for three different L_c, as shown in the figure S8. Therefore, the cancelling mutual inductance between two adjacent strips could be ignored when the coiled turns under 20 and L_c=20μm, because the ratio is small than 1%.

8. Substrate immunity

(A)

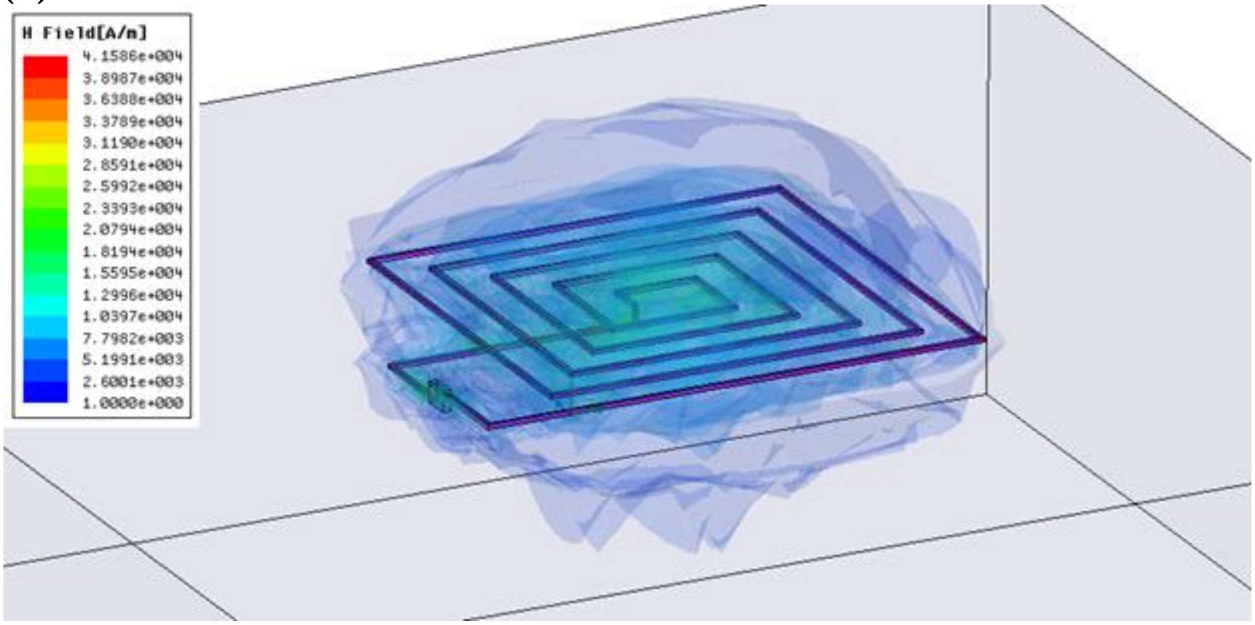

(B)

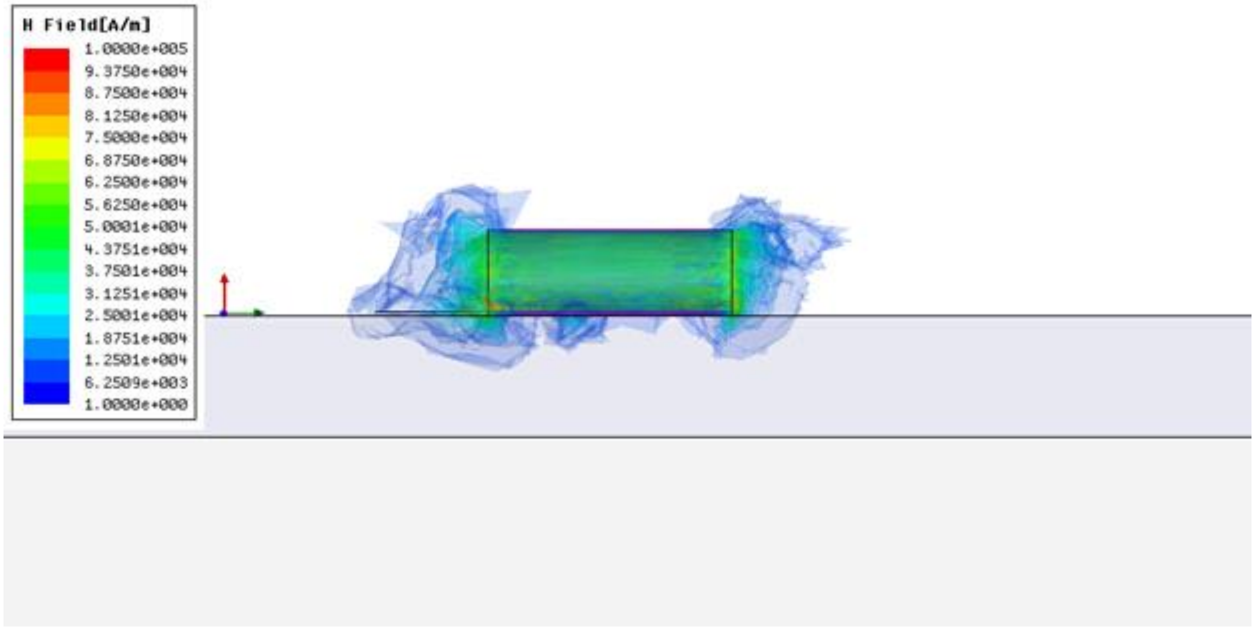

Figure S9: Magnetic field distribution of planar spiral inductor and tube inductor. (A) 5turns planar spiral inductor with 1µm by1µm rectangular cross section, 118µm outer diameter, 18µm inner diameter and 9µm line spacing. (B) 10 coiled turns tube inductor with 3.16 µm inner radius, 20 µm width, and 40nm SiN_x.

To better illustrate the substrate immunity of tube inductors, FEM simulation by HFSS was performed to compare the magnetic field distribution of a planar spiral inductor and a tube inductor on a silicon substrate, as shown in Fig. S9. The planar spiral inductor (Fig. S9(A)) has a 1μm by 1μm rectangular cross section, 118μm outer diameter, 18μm inner diameter and 9μm line spacing. The tube inductor (Fig. S9(B)) has an inner radius of with 3.16 μm, a width of 20 μm, and 10 coiled turns of a single conducting strip separated by a 40nm thick SiN_x membrane between turns.

In the simulation, the metal spirals were modeled as perfect conductors embedded in a SiN_x hollow-cylinder with dimensions specified above. The silicon substrate was meshed and the field was solved in the substrate too. The tube inductor structure with substrate underneath was placed in vacuum with radiation boundary condition in HFSS. Both the E field and H field met the boundary condition at the interface between the substrate and vacuum. All material properties including permeability, permittivity, and conductivity used in the model were set to constant values as shown in Table S2. Since the metal spirals were modeled as perfect conductor, the simulated data only contained information of inductance -- 1.8 nH for the spiral inductor and 0.19 nH for the 10-turn tube inductor at 5 GHz. Note that a smaller inductance value for the tube inductor was used to save computation time; however, to reach a similar inductance to that of the planar inductor, the tube inductor can be a structure with 10 strips connected in series, which gives the same penetration depth as a single strip structure simulated here. The fields were calculated when both devices fed with 1 Watt RF power. The simulated magnetic field value of the tube inductor and the planar inductor were set to the same minimum value ~1 A/m, in order to compare the penetration level of both structures. By plotting the magnetic field distribution @ 5GHz & 0º, we can compare the field penetration below the substrate and confinement above the substrate for both types of inductors, as shown in Fig. S9. The magnetic field H with 1A/m magnitude penetrate into the substrate about 5 μm for the tube inductor and 50 μm for the spiral inductor.

As can be seen clearly in Fig. S9, significant part of the magnetic field generated by the planar spiral inductor penetrates into the substrate, which will introduce serious substrate effects including capacitive effect and eddy current effect. In contrast, the magnetic field generated by the tube inductor is almost separated from the substrate, which means much less substrate parasitic effects are introduced.

Table S2 Material properties used in the FEM model

Material	Relative Permeability	Relative Permittivity	conductivity
SiN_x	7	1	0
Si	11.9	1	0

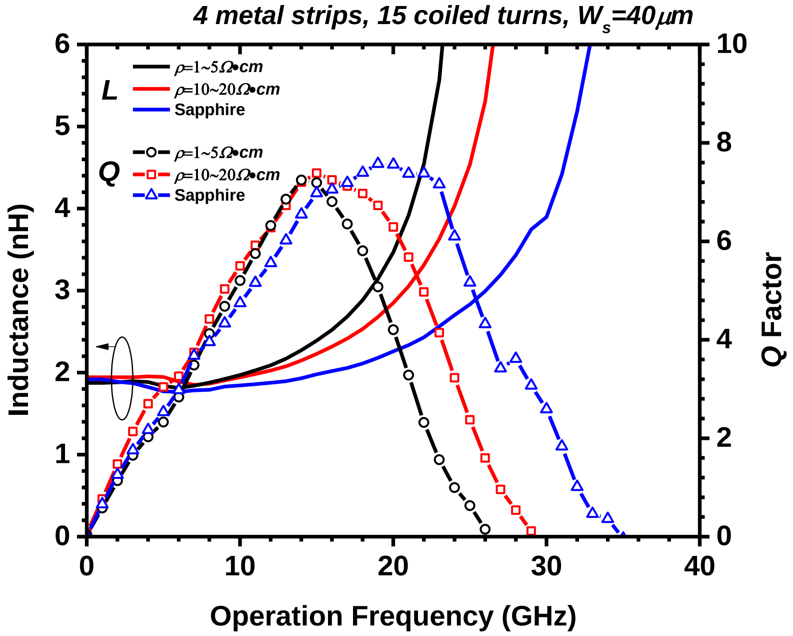

Figure S10: Experimental inductances (solid lines) and Q factors (symbol lines) with the same device structural configuration but on the different substrates are plotted to operation frequency. Black, red and blue curves are for p-Si (ρ=1~5Ω▪cm), p-Si (ρ=10~20Ω▪cm) and c-plane sapphire substrate.

On three different substrates, the tube inductors with the same device structural configurations demonstrate identical inductance values at low frequency range, but different values of Q_{max} and f_{Qmax}.

References

S1. Froeter, P. *et al*. 3D hierarchical architectures based on self-rolled-up silicon nitride membranes. *Nanotechnology* **24**, 475301 (2013).
S2. Goto, Y., Natsukari, Y. & Fujishima, M. New On-Chip De-Embedding for Accurate Evaluation of Symmetric Devices. *Jpn. J. Appl. Phys.* **47**, 2812-2816 (2008).
S3. Zhang, B., Xiong, Y. Z., Wang, L., Hu, S. & Li, J. L. W. De-embedding of On-Chip Inductor at Millimeter-Wave Range. *Jpn. J. Appl. Phys.* **51**, 086602 (2012).
S4. Huang, W. *et al*. On-Chip Inductors with Self-Rolled-Up SiN_x Nanomembrane Tubes: A Novel Design Platform for Extreme Miniaturization. *Nano Lett.* **12**, 6283-6288 (2012).
